# The Novel Halovirus Hardycor1, and the Presence of Active (Induced) Proviruses in Four Haloarchaea

**DOI:** 10.3390/genes12020149

**Published:** 2021-01-23

**Authors:** Mike Dyall-Smith, Friedhelm Pfeiffer, Pei-Wen Chiang, Sen-Lin Tang

**Affiliations:** 1 Computational Biology Group, Max-Planck-Institute of Biochemistry, 82152 Martinsried, Germany; fpf@biochem.mpg.de; 2 Veterinary Biosciences, Faculty of Veterinary and Agricultural Sciences, University of Melbourne, Parkville 3010, Australia; 3 Biodiversity Research Center, Academia Sinica, Nankang, Taipei 115, Taiwan; chiangpw@gate.sinica.edu.tw (P.-W.C.); sltang@gate.sinica.edu.tw (S.-L.T.)

**Keywords:** archaea, haloarchaea, temperate virus, *Haloferax*, *Haloarcula*, *Halorubrum*, halobacteria, pleolipovirus, caudovirus, siphovirus

## Abstract

The virus Hardycor1 was isolated in 1998 and infects the haloarchaeon *Halorubrum coriense*. DNA from a frozen stock (HC1) was sequenced and the viral genome found to be 45,142 bp of dsDNA, probably having redundant, circularly permuted termini. The genome showed little similarity (BLASTn) to known viruses. Only twenty-two of the 53 (41%) predicted proteins were significantly similar to sequences in the NCBI nr protein database (E-value ≤ 10^−15^). Six caudovirus-like proteins were encoded, including large subunit terminase (TerL), major capsid protein (Mcp) and tape measure protein (Tmp). Hardycor1 was predicted to be a siphovirus (VIRFAM). No close relationship to other viruses was found using phylogenetic tree reconstructions based on TerL and Mcp. Unexpectedly, the sequenced virus stock HC1 also revealed two induced proviruses of the host: a siphovirus (Humcor1) and a pleolipovirus (Humcor2). A re-examination of other similarly sequenced, archival virus stocks revealed induced proviruses of *Haloferax volcanii*, *Haloferax gibbonsii* and *Haloarcula hispanica*, three of which were pleolipoviruses. One provirus (Halfvol2) of *Hfx. volcanii* showed little similarity (BLASTn) to known viruses and probably represents a novel virus group. The *attP* sequences of many pleolipoproviruses were found to be embedded in a newly detected coding sequence, split in the provirus state, that spans between genes for integrase and a downstream CxxC-motif protein. This gene might play an important role in regulation of the temperate state.

## 1. Introduction

Viruses of prokaryotes are extraordinarily numerous in aquatic environments [1,2], commonly outnumbering cells by a factor of 5 to 10 [3]. They play significant roles in a variety of important biological and biogeochemical processes, including the lysis of cells and the consequent release of organic matter, selective sweeps of prokaryotic populations that drive the evolution of both virus and host, the acceleration of genetic exchange and the redirection of host metabolism [4]. They continue to provide a source of surprising new discoveries and insights, such as the BREX (BacteRiophage EXclusion) defense system [5] of bacteria and the viral counter defense to this based on the DNA mimic protein Ocr [6]. Another impressive example of their ingenuity is the recently described phage-specific peptide communication system, termed the arbitrium system, that allows proviruses to decide between lytic and lysogenic lifestyles [7].

Viruses of diverse morphotypes are known to infect extremely halophilic archaea (class *Halobacteria*), including caudoviruses such as phiH1 [8] and HF1 [9] (reviewed by [10,11,12,13]); spindle shaped viruses such as His1 [14,15,16]; lipid enveloped pleolipoviruses such as His2 [17,18] and the spherical/icosahedral, membrane-containing sphaerolipoviruses such as SH1 [19,20]. All possess DNA genomes, and many are temperate, with numerous related proviruses being found in the genome sequences of haloarchaea. While the packaged genomes of haloarchaeal caudoviruses are always linear dsDNA [13], they are derived from concatemeric intermediates that are either cut at sequence-specific sites resulting in unit-length genomes, such as in HF2 [21], or they are cut after head-full packaging, producing genomes that are circularly permuted and terminally redundant [13].

Sequencing technology is now sufficiently robust to allow the recovery of valuable information from archival biological material containing low amounts of DNA, and this was recently applied to haloviruses Serpecor1 and Hardycor2 [22]. Frozen stocks of these viruses had been stored since 1998 and although no longer viable, their genomes could be sequenced and compared to other viruses, revealing they were related to the caudovirus HF1 and similar viruses of the recently approved genus *Haloferacalesvirus* [9,12].

In this study, the archival virus stock of Hardycor1 (originally labelled HC1) was analysed to determine its viral genome sequence, genetic composition and relationship to other known viruses. Hardycor1, like Hardycor2, was recovered in 1998 from Lake Hardy, a hypersaline lake in Victoria. It gave clear plaques on lawns of *Halorubrum coriense.* Based on its genome, Hardycor1 is a novel siphovirus, probably representing a distinct genus. Surprisingly, the virus stock also contained two induced proviruses of *Hrr. coriense*, and this was found to be a common phenomenon in other halovirus stocks. A stock from the model haloarchaeon *Hfx. volcanii* contained two induced proviruses, one being a previously unrecognized provirus (Halfvol2) that may represent a novel virus genus.

## 2. Materials and Methods

### 2.1. Virus Isolation

The methods have been described recently in [22]. Briefly, a hypersaline water sample, collected in 1998 from Lake Hardy, Victoria (35° 04′ S, 141° 44′ E), was used to isolate viruses on soft-agar lawns of *Hrr. coriense* Ch2^T^ (DSM 10284) incubated at 37 °C. A clear plaque was picked, purified 3 x by further titrations, and then stored in HF diluent [23] at −80 °C. The stock was labelled HC1.

### 2.2. DNA Isolation, Sequencing and Assembly

The strategy and methods have been described previously [22]. Briefly, the virus stock was processed to extract DNA, which was then amplified using the Qiagen REPLI-g Mini Kit in order to achieve sufficient material for next-generation sequencing. One microgram of the qualified DNA was sequenced at Yourgene Health Co. (Taipei, Taiwan) using the Illumina platform (HiSeq 2500 sequencer; 2 × 250 bp paired-end reads). Assembly of Illumina reads was performed using the de novo assembler within Geneious version 10.2.6 [24,25].

### 2.3. Bioinformatics Analyses

For gene annotation, a combination of gene prediction with GeneMarkS-2 [26] and manual refinement using database searches (BLASTp/BLASTn) was used. The gene calling program Glimmer3 [27], available within the Geneious Prime environment, was used to confirm the GeneMarkS-2 predictions of genes *hrrhc1_050* and *hrrhc1_095*. Dot plot comparison used the YASS alignment tool [28], available via the webserver [29]. Genome comparisons were performed using the GeneWiz browser 0.94 [30]. CRISPR spacer searches used the IMG/VR spacer BLAST tool [31], or the BLAST CRISPRs tool [32]. VIRFAM typing of head-neck-tail proteins was performed using the webserver at [33,34]. Transmembrane domain and signal sequence prediction used Phobius at [35,36].

## 3. Results

### 3.1. Isolation and Sequence

Upon isolation in 1998, Hardycor1 produced 2–3 mm clear plaques on lawns of *Hrr. coriense*. In 2015, DNA from a stored stock of this virus (labelled HC1) was extracted and sequenced. Read assembly produced a high coverage contig of 45,142 bp in length, and circular in form. As shown later, the most parsimonious interpretation of the circular assembly is that the virus genome is packaged as linear dsDNA molecules with ends that are terminally redundant and circularly permuted. A summary of the sequencing results is shown in Table 1. 

The %G + C of the host, *Hrr. coriense*, is 66.6% [37], very close to that of the virus. Contig sequences from contaminating DNA in the same virus sample exactly matched the genome of *Hrr. coriense*, confirming the host.

BLASTn searches against the GenBank nr/nt nucleotide database (E-value ≤ 10^−15^, March, 2020) returned hits to only two short (~400–500 bp) regions of the Hardycor1 genome, and one of these (nt 39800–40246) was non-specific to highly repetitive eukaryotic sequences. The other region, of 436 bp (nt 25604–26035), matched sequences from two haloarchaeal siphoviruses, HCTV-1 and HHTV-2, at 66–69% nucleotide identity.

Analysis of tetramer frequencies revealed the absence of three tetrameric sequences, all of which are palindromic (Table 2). Another three non-palindromic tetramers were strongly avoided. A similar analysis of palindromic 6-mers (excluding those with absent or under-represented tetrameric cores shown in Table 2) found that the viral genome lacks 22 such motifs (Table 3). The results are indicative of a strong selection against numerous 4–6 bp sequence motifs, particularly palindromic motifs. Most likely, this helps to avoid host defences such as restriction-modification (R-M) systems. The host species, *Hrr. coriense*, has five annotated genes involved in R-M (Supplementary Table S1) and has previously been shown to be *dam*-methylated [38].

Repeat sequences: A number of related repeats of varying length (20–83 bp) occur in several intergenic regions, upstream of six ORFs (HrrHc1_045, _095, _160, _230, _235 and _245). One of these repeats partly overlaps the start of three CDS (HrrHc1_045, _95 and _235) with the result that the predicted proteins have identical N-termini (MNANT...). There is also a 530 bp direct repeat (nt 21,857–22,916) that spans the borders of three CDS (HrrHc1_135, _140 and _145), with the latter two predicted proteins sharing 106 aa of identical N-terminal sequence.

**Figure 1:**
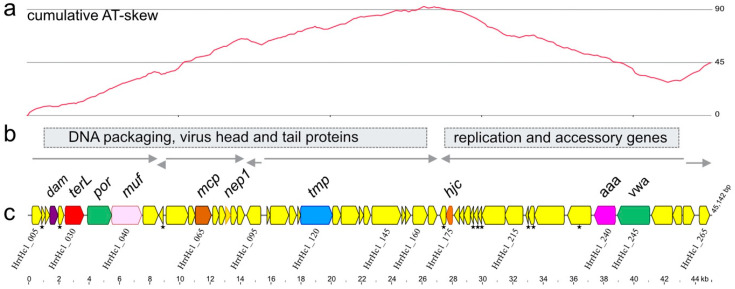
(**a**) Cumulative AT-skew plot (window = 45 nt) of Hardycor1 genome. (**b**) Functional modules and predicted transcription directions. (**c**) Genome map of Hardycor1. Gene names and the proteins they specify: *dam*, Dam methylase; *terL*, large subunit terminase; *por*, portal protein; *muf*, MuF-family head morphogenesis protein (SSP1 gp7 family); *mcp*, major capsid protein; *nep1*, neck protein of type 1; *tmp*, tape measure protein; *hjc*, Holliday junction resolvase Hjc; *aaa*, AAA ATPase; *vwa*, Von Willebrand factor type A (vWA) interaction domain that includes a metal ion-dependent adhesion site (MIDAS). Asterisks immediately below gene arrows indicate the predicted hypothetical proteins contain CxxC motifs. The locus tags (e.g., HrrHc1_005) of several genes are shown below the gene map. Scale at bottom represents DNA length in kb.

Annotation of the genome sequence revealed 53 CDS, and the map displayed in Figure 1 represents the unit genome in linear form with the starting base chosen for its proximity to the large subunit terminase gene, *terL* (*hrrhc1_030*) but placed upstream of the five closely spaced genes that precede *terL* because they were in the same orientation, had overlapping CDS and are likely to be transcribed together. Genes are generally closely spaced, with 27 genes (50%) overlapping at their start and stop codons, and a further 13 genes (24%) separated by 10 bp or less. Most genes are oriented inwards to a point around 27 kb (Figure 1b,c). This broad organizational pattern is reflected in the cumulative AT-skew plot shown above (panel a), which displays a major inflection at this point, and falls steadily to either side except for short regions corresponding to local reversals in gene orientation.

The predicted Hardycor1 proteome was submitted to VIRFAM [34], which classified four of the inferred proteins as caudovirus homologs (TerL, Portal, MCP and Nep1) and predicted Hardycor1 was most likely a siphovirus.

A BLASTn search of the GenBank nr/nt nucleotide database restricted in scope to sequences from Halobacteria (taxid:183963) + Viruses (taxid:10239), and at a reduced stringency than before (E-value ≤ 10^−10^), returned two short matches (Table 4), one of which was previously mentioned. These were to *tmp* (*hrrhc1_120*), the gene encoding the tape measure protein, and *hrrhc1_160*, which specifies a hypothetical protein. The top match for *tmp* was to a 540 bp region within an annotated tape measure protein gene (HPS36_14875) carried on the chromosome of *Halorubrum* sp. strain RHB-C. The top match to *hrrhc1_160* was the halovirus HCTV-1 gene DNAM5_77. HCTV-1 is a siphovirus infecting *Har. californiae* [12]. 

**Figure 2:**
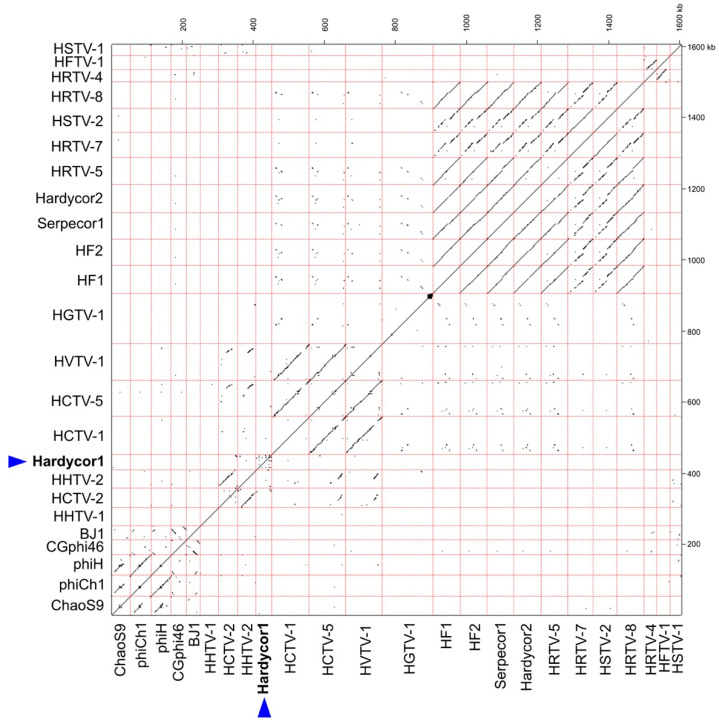
Dot plot comparison of the Hardycor1 genome with the genomes of 21 other tailed haloviruses. Nucleotide sequence similarity analysis was performed using YASS (BLASTz; E-value threshold = 10^−10^) [28]. Virus names are shown along the left and lower axes, and genome borders are shown as pink dashed lines. The position of Hardycor1 is indicated by a blue triangle. Sequence accessions for each halovirus are given in Figure 3. The scale is shown in kb along the upper and right axes.

Nucleotide sequence similarity of Hardycor1 with the genomes of 23 known tailed haloviruses is presented as a dot plot in Figure 2. Related viruses are clearly detected as lines of similarity parallel to the main diagonal, such as members of the *Myohalovirus* genus (ChaoS9, phiCh1 and phiH1; lower left corner), and members of the *Haloferacalesvirus* genus (HF1 to HRTV-8; near upper right corner). Hardycor1 (blue triangle) shows little or no sequence similarity to any of the other haloviruses.

**Figure 3:**
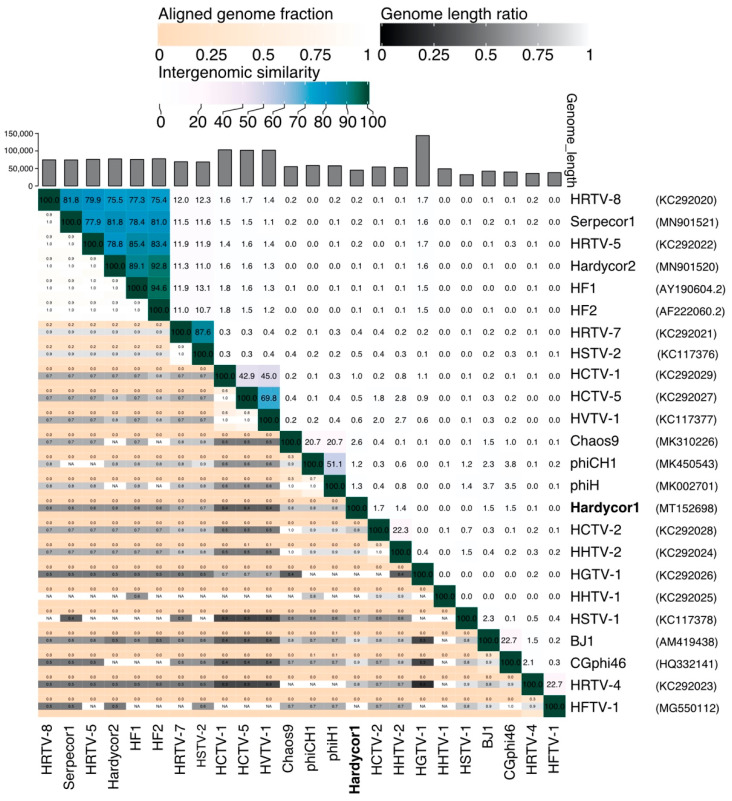
Intergenomic similarity of haloviruses (upper right triangle) based on whole genome sequences and calculated using the VIRIDIC webserver. Both colour scale (top) and numeric values are displayed. Lower left triangle displays aligned genome fraction and genome length ratio (colour scales shown at top). Genome lengths are indicated by vertical bars at top edge. Virus names and GenBank accessions are shown at right edge.

The low nucleotide sequence similarity of Hardycor1 to other tailed haloviruses prevents any meaningful alignment or phylogenetic inferences; however, whole genome similarity values are useful to define viral taxa. Figure 3 shows a heat map of intergenomic similarities of tailed haloviruses, produced using the VIRIDIC suite of programs [40]. Values are calculated using the traditional algorithm recommended by the International Committee on Taxonomy of Viruses (ICTV), Bacterial and Archaeal Viruses Subcommittee. In this scheme, members of the same species share ≥95% nt similarity, while members of the same genus share more than about 70% nt similarity, although more recently, the ICTV have suggested a threshold of ~50% nt similarity for caudoviruses [41]. Hardycor1 shows negligible similarity (0–2%) to the other 23 virus genomes and represents a novel species and genus. An independently described algorithm, VICTOR [42], calculates similarities of viral genomes based on nucleotide or protein sequences, and in both cases, Hardycor1 was predicted to represent a novel species and novel genus (Supplementary Table S2).

### 3.2. Annotation and Predicted Proteins

Twenty-two of the 53 annotated proteins (41%) returned significant matches (BLASTp, E-value ≤ 10^−15^) to protein sequences of the NCBI nr database, and the top matches are shown in Table 5. Fourteen matched the proteins of various species of haloarchaea, six matched proteins of three haloviruses (HCTV-1, HHTV-1 and HCTV-2), and two matched bacterial proteins. The three haloviruses with similar proteins are all siphoviruses with linear, circularly permuted dsDNA genomes, and infect species of *Haloarcula* [12,13].

The presence of conserved protein domains and characteristic VIRFAM profiles of virus proteins [34] allowed functional assignments for eight proteins, revealing that the first 27 kb of the Hardycor1 genome carries genes encoding key proteins of caudoviruses, including the large subunit terminase (TerL), portal protein (Por), major capsid protein (Mcp) and tape measure protein (Tmp). A *muf* gene is found just downstream of the portal protein gene (*por*), and specifies a MuF (SPP1 gp7) family protein of the longer type [43]. MuF proteins have been reported to have a number of functions in different viruses, such as protecting the ends of viral DNA from nuclease attack when entering a host cell. The close gene spacing and typical arrangement of viral genes identified this region as being responsible for DNA packaging, virus assembly and morphogenesis. The absence of genes for tail-sheath or base-plate J proteins is consistent with the VIRFAM prediction that Hardycor1 is a siphovirus. Upstream of *terL* is a dam gene (*hrrhc1_020*) encoding a putative N-6-adenine-methyltransferase (Dam).

A strongly conserved feature among caudoviruses is a pair of genes upstream of the tape measure protein gene (*tmp*) that encode two related chaperone proteins via programmed ribosomal frameshifting [44,45]. In Hardycor1, these correspond to HrrHc1_110 and HrrHc1_115, and a classical −1 slippery sequence of the type X XXY YYZ is found at the appropriate position near the end of HrrHc1_110 (nt 17589–17595; G GGA AAT) that would allow translation of a protein encompassing the CDS of both genes. A similar protein to HrrHc1_110 is found in *Natronolimnobius* (NGM69196.1; 34% aa identity), is encoded by a gene upstream of a tape measure protein gene and also contains a classical –1 slippery sequence near its 3′ end (G GGA AAG, nt 226569–226575, AAKXY010000003.1). The inferred tape measure protein (HrrHc1_120) of Hardycor1 is 703 aa long, and would predict a tail length of about 84 nm using the formula described by [46].

Genes *hrrhc1_050* and *hrrhc1_095* are unusual, as they are found on the complementary strand to the other genes in this region (Figure 1b,c). However, both are predicted by GeneMarkS2 and by Glimmer3 (see Methods), and both proteins have features consistent with other haloviral proteins. For example, HrrHc1_050 protein contains two CxxC motifs [22] and a predicted C-terminal membrane spanning domain, and HrrHc1_095 protein has a pI of 4.24 and an over-abundance of Asp residues, typical features of haloarchaeal proteins [47]. Manual examination of alternative ORFs in the regions of HrrHc1_050 and HrrHc1_095 did not reveal any that were more likely.

The right end (27–45 kb) consists largely of genes specifying proteins of unknown function (yellow in Figure 1), even though seven of these are similar to proteins of haloarchaea or viruses (Table 5). All but the last two genes face inwards, an organisation similar to that of bacterial siphoviruses [48]. Like many siphoviruses, this region includes a gene specifying a Holliday junction resolvase Hjc (HrrHc1_175), an endonuclease that among other roles acts in debranching DNA structures to allow packaging of the viral genome into capsids [49,50].

Two other proteins specified by genes in the replication/accessory module of the genome have conserved functional domains. The 711 aa protein HrrHc1_245 is predicted to carry a von Willebrand factor A domain (vWA) and a metal ion-dependent adhesion site (MIDAS) domain (Table 5). Such domains often function in protein–protein interactions [51]. The encoding gene is situated next to a gene encoding an AAA ATPase (HrrHc1_240), an arrangement that is commonly found in bacteria and archaea [52,53]. Similar vWA domain proteins have been reported previously in siphoviruses of haloarchaea: HCTV-2, HHTV-2 and HVTV-1 [11,12,13]. In the case of HVTV-1, the corresponding gene is also near to a gene for an AAA ATPase, and in the same orientation. The close proximity of genes encoding an AAA ATPase and a vWA-MIDAS domain protein has been reported in thermophilic archaeal viruses, such as Acidianus Two-Tailed Virus (ATV) [54]. The interaction between AAA ATPase and a vWA-MIDAS domain protein has been closely studied in several cases, and a common finding is that the vWA-MIDAS domain protein provides an adaptor function, while the AAA ATPase acts as a chaperone [55,56].

Four annotated proteins have predicted transmembrane domains (TMD) or a signal sequence (HrrHc1_050, _150, _155 and _165). Ten hypothetical proteins contain one or more CxxC motifs (asterisked in Figure 1c), a signature feature of zinc-finger domain proteins [57]. HrrHc1_050 is a 104 aa long, CxxC motif containing protein that carries a strongly predicted TMD near its C-terminus. The gene encoding this protein is located on the minus-strand, unlike the other genes around it (Figure 1). HrrHc1_150 has a predicted signal sequence, and is the only annotated protein to do so. HrrHc1_155 possesses three evenly spaced TMDs (Phobius) and shares this and other similarities with the well-studied S105 holin of lambda [58,59]. HrrHc1_150 and HrrHc1_155 are separated by only 11 bp. The fourth protein, HrrHc1_165, has a TMD near its N-terminus, but this is not predicted to be a signal sequence. The gene is located just before the major switch in coding strand that occurs around 27 kb (Figure 1), and its inferred protein matched several similar sequences in the NCBI nr protein database, although none have an annotated function.

### 3.3. Protein-Based Phylogenetic Analyses

**Figure 4:**
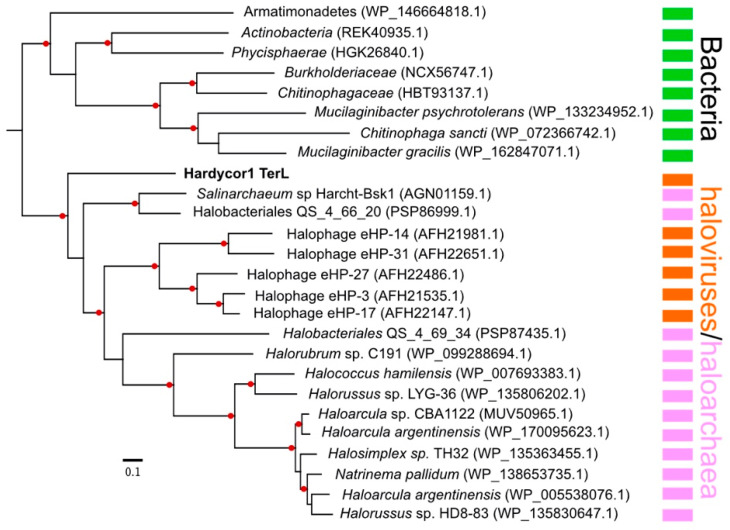
Phylogenetic tree reconstruction of Hardycor1 TerL protein. The top 100 BLASTp matching proteins were downloaded from the NCBI nr protein database, partial sequences and duplicates removed, and the remaining sequences imported into Geneious and aligned (MAFFT aligner). Trees were inferred using the FastTree algorithm (approximately maximum likelihood) within Geneious and default settings. A consensus tree (100 repetitions) was generated, with branch support values ≥ 80% indicated by red discs. Only part of the full tree is shown, the section that includes Hardycor1 TerL and closest related proteins. Genbank accessions are shown in brackets. Scale bar (lower left) represents 0.1 expected substitutions per site. Coloured bars indicate major taxonomic groups: green, bacteria; orange, haloviruses; pink, haloarchaea (class Halobacteria).

The large subunit terminase (TerL) is highly conserved in caudoviruses and has often been used to infer phylogeny [60,61,62]. The Hardycor1 TerL sequence showed significant similarity to numerous homologues present in the NCBI nr protein database. A phylogenetic tree reconstruction is presented in Figure 4 and shows that the Hardycor1 protein clusters with TerL sequences of haloarchaea and haloviruses but is distinct and branches just outside the other members of this clade.

The major capsid protein is also commonly used to infer viral phylogeny [63], but the Hardycor1 Mcp shows low similarity to known homologs (≤31% aa identity), and the top four BLASTp hits were to a wide variety of organisms, including an oceanic (bacterial) virus (QDP55370.1) and three diverse taxa of bacteria (*Pseudomonas*, *Bacteroidetes*, *Paenibacillus*). Without more examples of specifically related relatives, no meaningful phylogenetic inferences are possible based on Mcp trees. 

**Figure 5:**
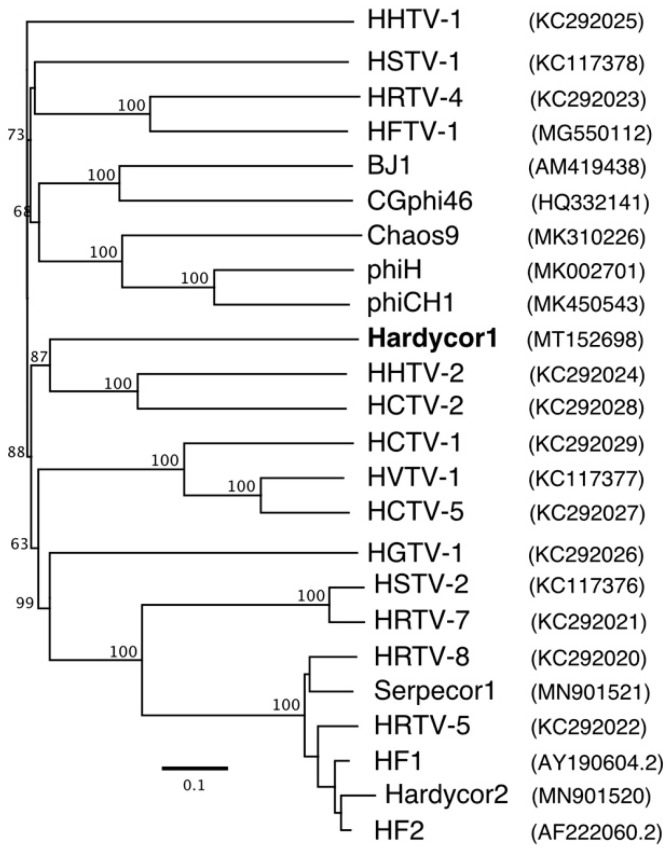
Phylogenetic tree reconstruction of tailed haloviruses inferred from viral proteomes using the Genome-BLAST Distance Phylogeny method (GBDP) under optimal settings (formula VICTOR d6), as implemented at the DSMZ webserver [42,64]. Percentage support values above 60% are shown near the branch points. The branch lengths are scaled in terms of the GBDP distance formula d6 [42]. Tree scale (0.1) is indicated by the bar. Accessions are given at the right in brackets.

A whole proteome-based phylogenetic reconstruction is presented in Figure 5, and shows Hardycor1 branches deeply and is not closely related to other known tailed haloviruses.

### 3.4. Match to CRISPR Spacer

The Hardycor1 genome was used to search for CRISPR spacer matches at the IMG/VR and CRISPRfinder websites (accessed December 10, 2020; see Methods). Only one significant match was found (Supplementary Table S3), to a 40 nt region (nt 17635–17674) found between the two annotated CDS immediately upstream of the gene encoding tape measure protein (*tmp*). The source of the spacer sequence was a halite endolithic microbial community found in the Atacama Desert, Chile [65].

### 3.5. Active Proviruses of Hrr. coriense

**Figure 6:**
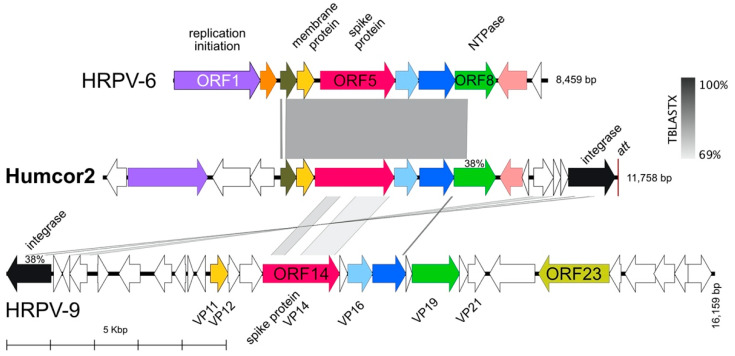
Provirus Humcor2 (MW344764) compared to the alphapleolipovirus HRPV-6 (JN882266) and the betapleolipovirus HRPV-9 (KY965934). Genes coding for corresponding proteins are coloured the same. Similarity, tBLASTx is indicated by greyscale shading, with the greyscale key shown at the top right. Length scale (kb) is given at the lower left.

Assembly of sequence reads of the Hardycor1 virus stock (HC1) revealed another circular contig, distinct from Hardycor1, that was 11,758 bp in length with a high read coverage, and matched a region on contig 20 of the *Hrr. coriense* draft genome (Table 6). The circular nature of this contig indicated it was an extrachromosomal element and not simply an amplified fragment of host chromosomal DNA. Its gene content indicated it was a virus, closely related to pleolipoviruses such as HRPV-6 (Figure 6), and was designated Humcor2. 

A map of Humcor2 is shown in Figure 6. It begins just after the 3′ end of tRNA-Pro gene C464_t04328 and ends after the 13 bp *att* sequence, which is identical to the 3′ end of the same tRNA. Near the *att* sequence is a gene coding for an integrase, a typical pattern for integrative prokaryotic viruses, including haloviruses [10,66]. Currently, the only available genome sequence of *Hrr. coriense* is a draft consisting of 69 contigs (accession GCF_000337035 [67], and a nucleotide alignment with Humcor2 revealed it to be identical to *Hrr. coriense* except for three separate 90 bp long artifactual direct repeats in the *Hrr. coriense* draft genome sequence, most likely assembly errors due to poor quality reads. Many of the predicted proteins of Humcor2 are similar to those of alphapleolipoviruses, such as HRPV-6 (Halorubrum pleomorphic virus 6) and its close relative HRPV-2 (Halorubrum pleomorphic virus 2) [17]. In summary, Humcor2 represents the extrachromasomal form of a pleolipoviral provirus, and most likely originates from virions. Both alpha and beta pleolipoviruses have circular dsDNA genomes [17].

**Figure 7:**
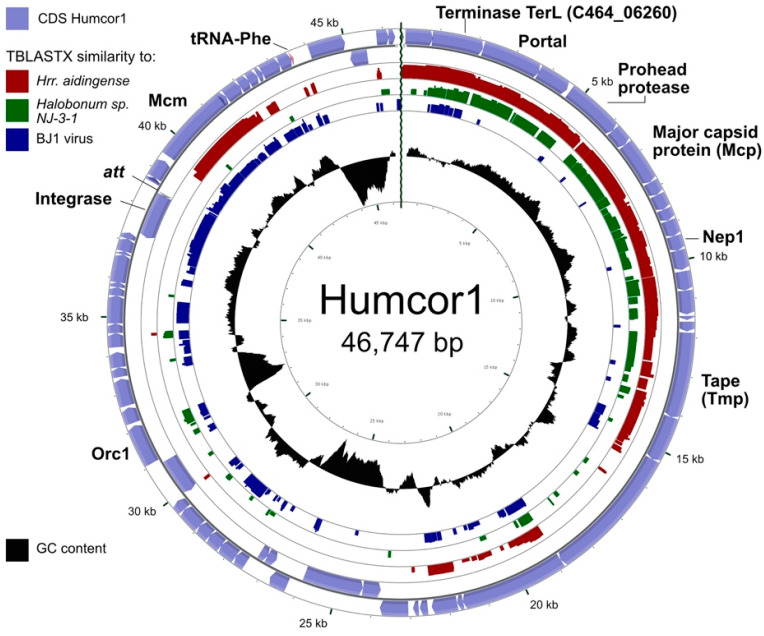
Provirus Humcor1 compared to provirus elements of *Hrr. aidingense* (AOJI01000015, contig_15, nt 8804–39413), *Halobonum* sp. NJ-3-1 (CP058579, nt 3124623–3124210) and halovirus BJ1 (AM419438). The outer, light blue rings show the annotated CDS of Humcor1, with the coding strand indicated by the arrow direction. The predicted protein products of several genes are given, with names shown next to their encoding gene. For more details see the text. Similarity (tBLASTx; E-value ≤ 10^−10^) to the two proviruses and halovirus BJ1 are shown as inner, coloured rings, with the colour key displayed at the top left. Below the similarity rings is a plot of GC content (black), with higher than average GC content depicted as outward pointing peaks, and lower than average GC content as inwardly pointing peaks. The scale, in kb, is shown at the outer perimeter.

Humcor1 (Table 6) is a second provirus of *Hrr. coriense* that was found in the assembled sequence reads from a different archival virus stock, labelled CC1. This virus isolate was recovered from Cheetham saltern (38° 09’ 23.5”S 144° 25’ 41”E) in 1998 and infected *Hrr. coriense* Ch2^T^. The provirus Humcor1 present in this stock assembled as a circular contig (Figure 7) and carries many genes that are characteristic of caudoviruses including genes encoding large subunit terminase (TerL), portal protein (Por), major capsid protein (Mcp) and tape measure protein (Tmp). No genes for tail sheath, base plate or tail fibres were detected, so it is most likely of the siphovirus type, as supported by VIRFAM typing of the head proteins (see Methods), which predicted Humcor1 as a siphovirus and also identified the Nep1 homologue (HK97 gp10 family phage protein) as C464_06210. Related proviruses are found in the genome sequences of *Hrr. aidingense* JCM 13560 and *Halobonum* sp. NJ-3-1 (red and green rings of Figure 6). The most closely related halovirus is BJ1 (Figure 7, blue ring), which shared a similar large subunit terminase (53% aa identity) as well as many of the accessory genes, such as an integrase, MCM and a strongly similar (82% aa identity) homologue of the hypothetical protein C464_06065 (nt 34838–35560). The circular assembly of Humcor1 is most likely due to head-full packaging of the virus genome, which produces a population that is circularly permuted and terminally redundant. This is also the case with halovirus BJ1 [68].

### 3.6. Proviruses Present in Virus Stocks from Other Haloarchaeal Hosts

Searches for proviruses were made using the sequence data collected from six other archival virus stocks that, like HC1, had been stored frozen since 1998 and analysed by sequencing in the same manner (see Methods). Four active proviruses were detected (Halfgib1, Harhisp1, Halfvol1 and Halfvol2) that matched chromosomal regions of *Hfx. gibbonsii* Ma2.38^T^, *Haloarcula hispanica* Y27^T^ and *Haloferax volcanii* DS2^T^ (Table 6). All assembled to circular contigs with high read coverage. Three are pleolipoviruses, and the fourth (Halfvol2) represents a novel virus group, and was previously unsuspected in the *Hfx. volcanii* genome. 

Halfgib1 is found to be integrated near the end of tRNA-Arg (C454_t15621) in the *Hfx. gibbonsii* Ma2.38^T^ genome. The Halfgib1 sequence aligned near perfectly to the draft *Hfx. gibbonsii* genome sequence, except that the draft genome sequence across this region contains two separate 90 bp direct repeats, most likely due to misassembly. Similar errors were mentioned earlier in the *Hrr. coriense* draft genome, which was part of the same sequencing study [69]. The closely related ARA6 strain of *Hfx. gibbonsii* has no integrated provirus at this tRNA. In *Hfx. volcanii* DS2, there is a 12.5 kb provirus present in the corresponding tRNA (CP001956.1, nt 1294959–1307485), as reported previously [70,71]. We denote this as Halfvol3, and it shares high (>90%) nucleotide similarity with Halfgib1 (Supplementary Figure S1) and both encode predicted proteins that show similarity to proteins of pleolipoviruses [17]. 

Harhisp1 assembled as a circular contig from reads recovered from halovirus stock HH1, which was produced from *Har. hispanica*. The contig matched a provirus integrated at tRNA-Ala (HISP_14435) of the host chromosome and encompassed genes hisp_14430 to hisp_14315. This region had previously been identified as being related to betapleolipoviruses, such as HHPV3 (see Figure 6 of [72]), and its excision from the chromosome had been detected using PCR amplification across the predicted *attP* region [71]. 

Halfvol1 and Halfvol2 were recovered as circular contigs from sequence reads derived from the virus stock HV2, which was produced from cells of *Hfx. volcanii*. Their summary characteristics are shown in Table 6. Halfvol1 matched one of two proviruses on the *Hfx. volcanii* chromosome that had been pointed out in earlier studies [70,71], and is found to be integrated at the tRNA-Pro gene (HVO_3017). It is affiliated with the betapleolipovirus group, and a closely related but smaller provirus (14,675 bp) is found to be integrated in the corresponding tRNA-Pro of *Hfx. volcanii* strain SS0101 (VMTR00000000.1, Supplementary Figure S2).

**Figure 8:**
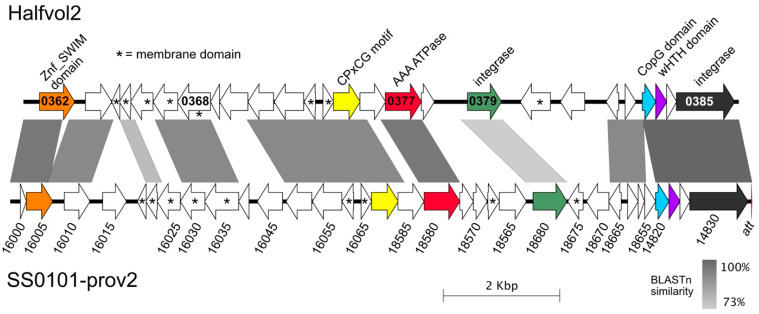
Comparison of Halfvol2 with a closely related provirus of *Hfx. volcanii* strain SS0101. Locus tag numbers are given inside gene arrows for Halfvol2 without the prefix (HVO_). The *Hfx. volcanii* SS0101-prov2 region was assembled from the following (overlapping) contigs: NZ_VMTR01000276, NZ_VMTR01000271, NZ_VMTR01000159 and NZ_VMTR01000131. Locus tag numbers are given below gene arrows but without their prefix (FQA18_). Asterisks denote encoded proteins predicted to contain transmembrane domains (Phobius/TMPred).

Halfvol2 had not previously been recognised as a provirus because its encoded proteins do not show significant matches to known viruses. It was first reported at the 2019 Halophiles conference [73]. The genome size, circular form and the ten annotated proteins with predicted transmembrane domains (asterisked in Figure 8) suggest it may be a lipid enveloped virus belonging to a novel virus group. Related proviruses are found in other haloarchaea (Supplementary Figure S3), and an example of similar size (12,732 bp) found in *Hfx. volcanii* SS0101 is compared to Halfvol2 in Figure 8. It is integrated in the corresponding tRNA-Ala of that strain. Searches of the ArcPP proteome database [74] revealed that the proteins expressed from several genes of both Halfvol1 and Halfvol2 have been detected in *Hfx. volcanii*. For example, HVO_0271 of Halfvol1 (dataset PXD011015) corresponds to the virus structural protein VP4 of Halogeometricum pleomorphic virus 1 (HGPV-1), and was detected in enriched fractions of cell surface proteins (archaella/pilins) after partial purification by CsCl centrifugation [75].

We designate the remaining previously described provirus of *Hfx. volcanii* as Halfvol3 (CP001956, nt 1294959–1307485), and for convenience, it is also shown in Table 6. While most of the genes of Halfvol3 have been shown to be transcribed [76], only one of the predicted proteins (HVO_0143) has been detected in proteomic studies (datasets PXD006877, PXD009116 and PXD011056) available from the ArcPP database [74].

No contig matching the length and sequence of Halfvol3 was produced by de novo assembly of the HV2 reads, and mapping of reads to Halfvol3 revealed they were only present in a low number, resulting in patchy coverage. However, reads spanning the termini were present, indicating that the element can excise and circularise. As added support for these findings, we examined the publicly available sequence archives of previous genomic sequencing studies of *Hfx. volcanii* and also found reads traversing the joined termini of Halfvol1 and Halfvol3. Two examples are given in Supplementary Tables S4A,B and the accompanying Supplementary Figures S4–S6. In the case of Halfvol1, the read coverage of the provirus region was significantly higher than the read coverage outside of the provirus (Supplementary Figure S6), indicating a high level of induced virus in strain Hv1 (sequenced in the study of [77]). Reads traversing the circularised termini of Halfvol2 were not found. In a recent study on hypermotile mutants of *Hfx. volcanii*, the deletion of Halfvol3 was detected as a secondary genome alteration in one of the analysed strains [78].

### 3.7. A CDS Frequently Encompasses the attP Sequence of Pleolipovirus-Like Proviruses

**Figure 9:**
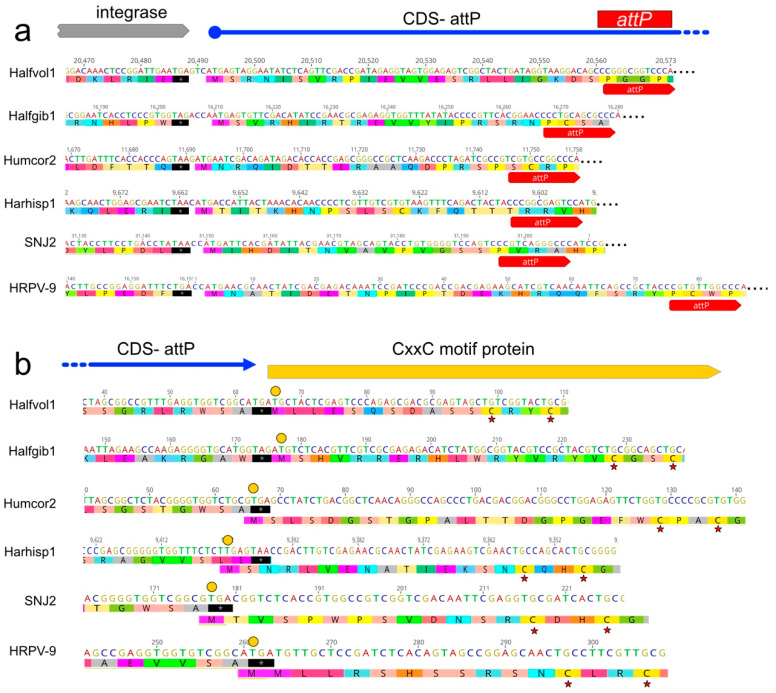
The *attP* sequence of many integrative pleolipoviruses are embedded in a CDS. Six examples are shown here: Halfvol1, Halfgib1, Humcor1, Harhisp1, SNJ2 and HRPV-9. (**a**) In each virus, the start codon of this CDS occurs near the end of the integrase gene. The translation (single letter code) and stop codon (black shading, with asterisk) are shown under the nucleotide sequence, and the position of *attP* is shown by the labelled red arrows. (**b**) The stop codon of the same CDS occurs near to, or overlaps with, the downstream CDS, which specifies a protein with two CxxC motifs. Start codons are indicated by yellow discs. Red asterisks point to the first CxxC motif.

Curiously, the *attP* sequences of the pleolipovirus-like proviruses described in this study are all found within a putative CDS that begins just downstream of the integrase gene and terminates adjacent to, or overlaps, the next CDS (a CxxC motif protein) in the circular form of their genomes (Figure 9). In all cases, the three adjacent CDS are on the same DNA strand, and so closely spaced that they may be transcribed together. A bridging CDS also occurs in the novel virus Halfvol2, as well as viruses and proviruses reported in earlier studies, such as SNJ2 and HRPV-9 (Figure 9). Although the lengths and inferred protein sequences of these genes vary, they regularly span the region between the genes for integrase and CxxC protein, genes that would be widely separated in the provirus state. Since the CDS is only complete when the virus genome is circularised, there is an obvious potential for switching off its activity upon provirus integration into the host genome.

## 4. Discussion and Conclusions

Hardycor1 was isolated 22 years ago and is a lytic halovirus infecting *Halorubrum coriense* strain Ch2^T^, but its genetic makeup was unknown until this study. It was found to have a 45,142 bp dsDNA genome encoding proteins that are typical of siphoviruses, a classification supported by gene organization, the presence of a *tmp* gene that predicts a tail length of 84 nm, and the absence of genes for tail sheath or baseplate J family protein (BpJ) [79]. This classification was confirmed by the conserved features of its head and neck proteins (VIRFAM). At the DNA sequence level, Hardycor1 shares little similarity with other described haloviruses, and standard comparisons show that it represents a novel species and genus. Inferred phylogenies using conserved proteins such as terminase (TerL) and the major capsid protein (Mcp) also support this conclusion.

The genome is most likely linear and packaged in a head-full manner that produces terminally redundant, circularly permuted ends. This was supported by protein similarities and protein phylogenetic tree reconstructions that indicated a distant relationship to haloviruses HCTV-2 and HHTV-2, both of which are siphoviruses with dsDNA genomes that are circularly permuted and terminally redundant [13]. Consistent with the view that Hardycor1 leads a lytic lifestyle, the genome does not carry a tRNA-like *attP* sequence or a gene for a site-specific integrase, and it lacks a gene for a DNA replicase. The absence of replicase genes means that the virus is dependent on host enzymes for this process, and in this respect, Hardycor1 is similar to HHTV-2 [12].

The viral genome has undergone strong selection against palindromic motifs, as evidenced by the absence of three tetrameric motifs (AGCT, CTAG and TGCA). This is a common finding among prokaryotic viruses [22], and protects the viral DNA from attack by sequence-specific defences of host species, such as restriction-modification (R-M) systems that are widespread in Halobacteria [80]. *Hrr. coriense* alone carries five genes predicted to encode R-M enzymes including a Dam methylase and two restriction endonucleases (Mrr) that target methylated DNA.

Three other haloviruses that infect *Hrr. coriense* have been described previously, the myoviruses HF2, Hardycor2 and Serpecor1 [9,22,81,82]. Like Hardycor1, their dsDNA genomes lack CTAG motifs and are under-represented in TGCA. Unlike Hardycor1, they lack the motif GATC and have the expected frequency of AGCT. Differences in the under-representation of palindromic (and non-palindromic) motifs seen in the genomes of viruses infecting the same host probably reflect distinct evolutionary histories, such as differing alternate host species, but could also be modulated by the defence systems carried by each virus. For example, DNA methylase genes are carried by Hardycor1 (*hrrhc1_020*), HF1 and Hardycor2 but not by Serpecor1 [22].

The overall pattern of gene organisation in Hardycor1 is typical of many siphoviruses [48]. Genes are generally oriented towards the centre, with a transition point at around 27 kb, and this divides the genome into two major regions that are functionally distinct. The left region carries genes for DNA packaging (TerL) and virus assembly (head and tail proteins). At the inner end of this region, near the major switch in gene orientation at 27 kb, there are three genes specifying proteins that contain transmembrane domains (*hrrhc1_155*, *hrrhc1_160* and *hrrhc1_165*), which could represent the lysis module. They occur in the corresponding region that holin and lysin genes are found in many siphoviruses [48], and which are usually transcribed late in the infection cycle. In bacterial viruses, these proteins are regulated so that cell lysis only occurs after virion assembly has been completed [59].

The right end of the genome (27–45 kb) is designated as the replication and accessory gene region but has many genes specifying proteins of unknown function. In well-studied siphoviruses, the corresponding region is transcribed early in infection and carries a variety of genes involved in the evasion of host defences, genome replication and the alteration of host metabolism to enhance virus production [48]. In Hardycor1, only a few genes code for proteins with conserved domains indicative of function, such as a Holliday junction resolvase (Hjc), an AAA ATPase and a von Willebrand factor type A (vWA) interaction domain protein that includes a metal ion-dependent adhesion site (MIDAS). Genes for Holliday junction resolvases are widespread in caudoviruses of bacteria [49,83], and occur in some haloarchaeal viruses, such as HCTV-2 [12], as well as other archaeal viruses [84]. Hjc functions to resolve recombination intermediates but can also debranch DNA prior to packaging as well as degrade host DNA [83,84].

The close association of genes for AAA ATPase and vWA-MIDAS proteins has been well documented [52], and examples are known among archaeal viruses such as Acidianus Two-Tailed Virus [54] and the haloarchaeal siphovirus HVTV-1 [11]. The functions of these proteins are unclear, but it is thought that vWA domain proteins interact with and assist the activity of AAA ATPases, which may function as chaperones [54].

Seven genes of the replication and accessory gene region code for proteins containing CxxC motifs, a signature feature of zinc-finger (ZF) domains that are commonly involved in interaction modules, such as DNA binding [85]. Such proteins are often small, commonly occur in haloviruses and are most frequently encoded by genes situated outside of the virus assembly module [22]. In *Hfx. volcanii*, small CxxC motifs containing proteins have been shown to be important in a variety of phenotypes, including stress adaptation, biofilm formation and swarming [86].

Two genes within the virus assembly module of the Hardycor1 genome are unusual (*hrrhc1_050* and *hrrhc1_095*), as they are found on the strand complementary to the other genes. This is uncommon, as the genes for head and tail proteins are typically closely spaced and all in the same orientation [48], allowing them to be transcribed together [81,82]. However, *hrrhc1_050* and *hrrhc1_095* are predicted independently by two gene callers (GeneMarkS2 and Glimmer3; see Methods), and the specified proteins have characteristics similar to other viral proteins. HrrHc1_050 contains two CxxC motifs [22,86] and a C-terminal membrane spanning domain, while the HrrHc1_095 protein has a low pI of 4.24 and an over-abundance of Asp residues, typical features of haloarchaeal proteins [47]. Alternative CDS options on the other strand are less likely. It is unclear why Hardycor1 is organised in this way. The haloarchaeal siphovirus HCTV-5 also has two CDS encoded on the opposite strand in the head and tail assembly module (HCTV5_113 and HCTV5_115).

The frequent presence of active proviruses in archival virus stocks was unexpected and facilitated the discovery of a novel and previously undocumented provirus (Halfvol2). The genome sequences of six induced proviruses were determined at high read coverage from five different virus stocks, and evidence for the presence of one more (Halfvol3) was detected using publicly available sequence read data. In the latter case, the increased read coverage of the virus sequence also indicated a high level of virus production. Three groups of viruses were identified: a siphovirus (Humcor1), five different pleolipoviruses and the novel virus Halfvol2. The gene content of Halfvol2 suggests it is probably lipid enveloped. In a previous study, the induction of specific proviruses in four species of haloarchaea was detected by PCR of *att* sequences [71], and evidence for the excision of Harhisp1 in *Har. hispanica*, as well as four others (in *Har. marismortui* and *Hmc. mukohataei*), was reported. In the current study, induced proviruses were not expected but were significant contaminants occurring in cell-free virus stocks, and their genomes were sequenced and assembled inadvertently.

In comparing the numerous proviruses described in this study, a surprising observation was the frequent occurrence of a CDS that overlaps the *attP* sequence, and which neatly spans between the viral integrase gene and a gene coding for a CxxC motif protein. This protein can only be produced after circularization of the virus genome and would not exist in the provirus state. The function(s) of the encoded protein will be interesting to elucidate, but its position and fragmentation upon integration into the host genome suggest it is intimately involved in the regulatory mechanisms underlying the transition between virulent and temperate states.

The frequency and multiplicity of active proviruses present in virus stocks highlight important issues when studying haloarchaea and their viruses. At the cell level, the interactions, mutations and phenotypes of haloarchaea may be influenced by provirus induction, loss or cross-infection. It is also unclear what perturbations in cell physiology might trigger provirus induction and subsequent virulent growth. To control for these variables, it would be prudent to document all functional proviruses of the species under study, and assess their activity when cells are placed under experimental conditions. As shown in this study, an unsuspected provirus was able to be detected by sequencing cell-free DNA preparations, such as viral lysates. On the other hand, when studying viruses, it is important to realise that one or more induced proviruses of the host may well be present, and could be difficult to remove from virus preparations using standard purification regimes. This would be most problematic if they share similar physical characteristics to the virus of interest, for example, if growing the pleolipovirus His2 on *Har. hispanica*, a host species known to produce the endogenous pleolipovirus Harhisp1.

## Figures and Tables

**Table 1:** Sequencing details for Hardycor1.

Virus	Host	Sequence Reads ^1^	Total Mb	Genome Length (bp)	G + C %	Read Coverage	Accession
Hardycor1	*Hrr. coriense*	17,097	21.6	45,142	67.8	95×	MT152698

^1^ Read length, 250 nt.

**Table 2:** Absent or under-represented tetramers in the Hardycor1 genome ^1^.

AGCT	CTAG	TGCA	CAGC	CATC	CCAG
0	0	0	0.01	0.03	0.11

^1^ Under-representation calculated as Odds Markov values where they are not zero.

**Table 3:** Absent palindromic 6-mers in the Hardycor1 genome ^1^.

First Base	6-mer Motifs Not Present in Hardycor1
A	ACATGT, AGATCT, AGCGCT, AGTACT, ATTAAT
C	CACGTG, CCATGG, CTCGAG, CTRYAG
G	GAATTC, GACGTC, GATATC, GCATGC, GGCGCC, GGTACC, GGGCCC, GRGCYC, GTATAC
T	TGGCCA, TGTACA, TTCGAA, TTTAAA

^1^ Excluding all motifs that include those listed as absent or under-represented in Table 2. All motifs have known restriction enzymes (REBASE, [39]).

**Table 4:** BLASTn matches to the Hardycor1 genome ^a^.

Hardycor1 Region (nt); Length	Hardycor1 Locus_Tag (Gene)	Matching Sequence (Accession)	Matched Region (nt); Name/Gene	Target Locus_Tag (Accession)	% Identity (E-Value)
18158–18692; 540 bp	*hrrhc1_120* *(tmp)*	*Halorubrum* sp. RHB-C (CP053941.1)	2930116–2930650; tape measure protein	HPS36_14875 (QKG94091.1)	66% (7 × 10^−28^)
25604–26035; 436 bp	*hrrhc1_160* (Hyp)	Halovirus HCTV-1 (KC292029.1)	43575–43153; hypothetical protein	DNAM5_77 (AGM11938.1)	69% (2 × 10^−21^)

^a^ June 10, 2020; BLASTn, default settings, nr nucleotide database.

**Table 5:** Annotated CDS of halovirus Hardycor1 (MT152698).

Start	Stop	Locus Tag	Length	Direction	Gene	Product	Protein Homologs ^a^
138	791	HrrHc1_005	654	+		hypothetical protein	E3374_RS16605 [*Halorhabdus* sp. H27]
788	1012	HrrHc1_010	225	+		CxxC motif protein	
1009	1323	HrrHc1_015	315	+		hypothetical protein	
1320	1883	HrrHc1_020	564	+	*dam*	probable Dam methylase	DJ70_12660 [*Halorubrum halodurans*]
1880	2260	HrrHc1_025	381	+		CxxC motif protein	
2337	3560	HrrHc1_030	1224	+	*terL*	large subunit terminase TerL	L593_06050 [*Salinarchaeum* sp. Harcht-Bsk1]
3809	5407	HrrHc1_035	1599	+	*por*	portal protein Por	FE783_12715 [*Paenibacillus mesophilus*]
5412	7382	HrrHc1_040	1971	+	*muf*	SPP1 gp7 family protein MuF	CMK96_05475 [*Pseudomonas* sp.]
7479	8501	HrrHc1_045	1023	+		hypothetical protein	
8506	8820	HrrHc1_050	315	–		CxxC motif protein	
8986	10,470	HrrHc1_055	1485	+		hypothetical protein	Natgr_1848 [*Natronobacterium gregoryi* SP2]
10,474	10,911	HrrHc1_060	438	+		hypothetical protein	
10,913	11,989	HrrHc1_065	1077	+	*mcp*	major capsid protein Mcp	IEX84_RS06545 [*Halarchaeum rubridurum*]
12,070	12,501	HrrHc1_070	432	+		hypothetical protein	
12,505	12,933	HrrHc1_075	429	+		DUF1073 domain protein	
12,935	13,288	HrrHc1_080	354	+	*nep1*	neck protein Nep1	G9C82_17265 [*Haloarcula* sp. R1-2]
13,285	13,710	HrrHc1_085	426	+		hypothetical protein	
13,707	14,201	HrrHc1_090	495	+		hypothetical protein	
14,337	15,266	HrrHc1_095	930	–		hypothetical protein	
15,676	15,879	HrrHc1_100	204	+		hypothetical protein	
15,883	17,115	HrrHc1_105	1233	+		hypothetical protein	AArcSl_1282 [*Halalkaliarchaeum desulfuricum*]
17,143	17,613	HrrHc1_110	471	+		hypothetical protein	G6M89_09280 [*Natronolimnobius* sp. AArcel1]
17,688	17,894	HrrHc1_115	207	+		hypothetical protein	
17,894	20,005	HrrHc1_120	2112	+	*tmp*	tape measure protein Tmp	C484_10631 [*Natrialba taiwanensis*]
20,007	20,552	HrrHc1_125	546	+		hypothetical protein	
20,554	21,759	HrrHc1_130	1206	+		hypothetical protein	BBD46_16545 [*Natrialba* sp. SSL1]
21,756	22,067	HrrHc1_135	312	+		hypothetical protein	
22,069	22,527	HrrHc1_140	459	+		hypothetical protein	
22,599	24,518	HrrHc1_145	1920	+		hypothetical protein	GS429_08425 [*Natronorubrum* sp. JWXQ-INN-674]
24,574	24,813	HrrHc1_150	240	+		hypothetical protein	
24,825	25,175	HrrHc1_155	351	+		predicted membrane protein	
25,290	26,255	HrrHc1_160	966	+		hypothetical protein	DNAM5_77 [HCTV-1], HHTV2_37 [HHTV-2]
26,324	26,917	HrrHc1_165	594	+		hypothetical protein	EPY72_RS18050 [*Halorussus* sp. LYG-36]
27,104	27,502	HrrHc1_170	399	–		CxxC motif protein	
27,499	27,936	HrrHc1_175	438	–	*hjc*	H-J resolvase ^b^ Hjc	BRC93_05600 [*Halobacteriales* archaeon]
28,034	28,390	HrrHc1_180	357	–		hypothetical protein	
28,390	28,674	HrrHc1_185	285	–		hypothetical protein	
28,671	29,117	HrrHc1_190	447	–		hypothetical protein	HHTV1_58 [HHTV-1]
29,114	29,314	HrrHc1_195	201	–		CxxC motif protein	
29,311	29,610	HrrHc1_200	300	–		CxxC motif protein	
29,607	29,867	HrrHc1_205	261	–		CxxC motif protein	
29,860	31,395	HrrHc1_210	1536	–		nucleic acid binding domain protein	HCTV2_73 [HCTV-2]
31,392	32,585	HrrHc1_215	1194	–		hypothetical protein	HCTV2_75 [HCTV-2], HHTV2_88 [HHTV-2]
32,774	32,938	HrrHc1_220	165	–		CxxC motif protein	
32,935	33,327	HrrHc1_225	393	–		CxxC motif protein	
33,324	35,321	HrrHc1_230	1998	–		hypothetical protein	DM826_07300 [*Halonotius* sp. F13-13]
35,523	37,079	HrrHc1_235	1557	–		CxxC motif protein	
37,288	38,694	HrrHc1_240	1407	–	*aaa*	AAA ATPase	HHTV2_10 [HHTV-2], HCTV2_83 [HCTV-2]
38,836	40,968	HrrHc1_245	2133	–	*vwa*	vWA and MIDAS domain protein	HCTV2_79 [HCTV-2], HHTV2_3 [HHTV-2]
41,082	42,461	HrrHc1_250	1380	–		hypothetical protein	
42,458	43,084	HrrHc1_255	627	–		hypothetical protein	
43,162	43,923	HrrHc1_260	762	+		hypothetical protein	
44,232	44,936	HrrHc1_265	705	+		hypothetical protein	

^a^ BLASTp searches (E-value ≤ 10^−15^, January 2021) against the NCBI nr protein database, with matches specified by their locus_tag followed by species or virus (in square brackets). Accessions for haloviruses HCTV-2, HHTV-2 and HHTV-1 are given in Figure 3. ^b^ H-J, Holliday Junction.

**Table 6:** Induced proviruses present in archival virus stocks ^a^.

Provirus	Length (nt)	Archival Virus Stock ^b^	G + C%	Read Coverage	Assembled Contig	Affiliation (Accession)	Comments
**Humcor1**	46,474	CC1	62.5	184	circular dsDNA	siphovirus (MW344765)	Matches *Hrr. coriense* Ch2^T^ (nt 170617–217091; AOJL01000026).
**Humcor2**	11,758	HC1	62.5	54	circular dsDNA	pleolipovirus (MW344764)	Matches *Hrr. coriense* Ch2^T^ (nt 11011–23038; AOJL01000020).
**Halfgib1**	16,280	HG1	56.5	470	circular dsDNA	pleolipovirus (MW344766)	Matches *Hfx. gibbonsii* Ma2.38^T^ (nt 269,983–286,444; AOLJ01000022).
**Harhisp1**	19,481	HH1	53.2	1403	circular dsDNA	pleolipovirus (MW344767)	Matches *Har. hispanica* Y27^T^ (nt 2722239 -2741719; CP006884)
**Halfvol1**	20,573	HV2	57.6	77	circular dsDNA	pleolipovirus	Matches *Hfx. volcanii* DS2^T^ (nt 231453–252025; CP001956)
**Halfvol2**	12,275	HV2	62.2	165	circular dsDNA	novel group	Matches *Hfx. volcanii* DS2^T^ (nt 329565–341853; CP001956)
**Halfvol3**	12,527	-	59.3	-	circular dsDNA	pleolipovirus	Matches *Hfx. volcanii* DS2^T^ (nt 1307486–1294960)

^a^ All from virus stocks except Halfvol3, which was found to excise using publicly available sequence data (see Section 3.6). ^b^ These virus stocks were described previously in [22].

## Data Availability

The virus and provirus sequences are available from the GenBank database (https://www.ncbi.nlm.nih.gov/genbank/). The Hardycor1 genome sequence has the GenBank accession MT152698. The proviruses Humcor2, Humcor1, Halfgib1 and Harhisp1 have the GenBank accessions MW344764, MW344765, MW344766 and MW344767, respectively.

## Supplementary Materials

The following are available online at https://www.mdpi.com/2073-4425/12/2/149/s1, Table S1: DNA methyltransferases and restriction endonucleases of *Hrr. coriense* Ch2^T^ (DSM 10284). Table S2: VICTOR prediction of taxonomic classification of tailed haloviruses. Table S3: CRISPR spacer match to Hardycor1. Supplementary Figure S1. Comparison of the genomes of Halfgib1 and Halfvol3 proviruses. Supplementary Figure S2: Comparison of the genomes of proviruses Halfvol1 and SS0101-prov1. Supplementary Figure S3: Gene Ortholog Neighbourhoods of Halfvol2 aligned using HVO_2248 of *Hfx. volcanii* DS2 as the reference. Table S4: Sequence reads spanning the circularized termini of the three chromosomal proviruses (Halfvol1, Halfvol2 and Halfvol3) of *Hfx. volcanii* DS2. Supplementary Figure S4: Screenshot of Geneious window showing Hfx. volcanii Hv90 reads (SRR11888928_Hv90) mapped to the termini of circularised Halfvol3. Supplementary Figure S5: Screenshot of Geneious window showing Hfx. volcanii Hv1 reads (SRX8436462) mapped to the termini of circularised Halfvol1. Supplementary Figure S6: Screenshot of Geneious window showing *Hfx. volcanii* Hv1 reads (SRX8436462) mapped to *Hfx. volcanii* DS2, showing the region around the left end of provirus Halfvol1 in the main chromosome.

## References

[1] Bergh O., Borsheim K.Y., Bratbak G., Heldal M. (1989). High abundance of viruses found in aquatic environments. Nature.

[2] Suttle C.A. (2005). Viruses in the sea. Nature.

[3] Hendrix R. (2002). Bacteriophages: Evolution of the majority. Theor. Popul. Biol..

[4] Coutinho F.H., Cabello-Yeves P.J., Gonzalez-Serrano R., Rosselli R., Lopez-Perez M., Zemskaya T.I., Zakharenko A.S., Ivanov V.G., Rodriguez-Valera F. (2020). New viral biogeochemical roles revealed through metagenomic analysis of Lake Baikal. Microbiome.

[5] Goldfarb T., Sberro H., Weinstock E., Cohen O., Doron S., Charpak-Amikam Y., Afik S., Ofir G., Sorek R. (2015). BREX is a novel phage resistance system widespread in microbial genomes. EMBO J..

[6] Isaev A., Drobiazko A., Sierro N., Gordeeva J., Yosef I., Qimron U., Ivanov N.V., Severinov K. (2020). Phage T7 DNA mimic protein Ocr is a potent inhibitor of BREX defence. Nucleic Acids Res..

[7] Erez Z., Steinberger-Levy I., Shamir M., Doron S., Stokar-Avihail A., Peleg Y., Melamed S., Leavitt A., Savidor A., Albeck S. (2017). Communication between viruses guides lysis-lysogeny decisions. Nature.

[8] Dyall-Smith M., Pfeifer F., Witte A., Oesterhelt D., Pfeiffer F. (2018). Complete genome sequence of the model halovirus phih1 (φh1). Genes.

[9] Tang S.L., Nuttall S., Dyall-Smith M. (2004). Haloviruses HF1 and HF2: Evidence for a recent and large recombination event. J. Bacteriol..

[10] Krupovic M., Forterre P., Bamford D.H. (2010). Comparative analysis of the mosaic genomes of tailed archaeal viruses and proviruses suggests common themes for virion architecture and assembly with tailed viruses of bacteria. J. Mol. Biol..

[11] Pietila M.K., Laurinmaki P., Russell D.A., Ko C.C., Jacobs-Sera D., Butcher S.J., Bamford D.H., Hendrix R.W. (2013). Insights into head-tailed viruses infecting extremely halophilic archaea. J. Virol..

[12] Sencilo A., Jacobs-Sera D., Russell D.A., Ko C.C., Bowman C.A., Atanasova N.S., Osterlund E., Oksanen H.M., Bamford D.H., Hatfull G.F. (2013). Snapshot of haloarchaeal tailed virus genomes. RNA Biol..

[13] Sencilo A., Roine E. (2014). A glimpse of the genomic diversity of haloarchaeal tailed viruses. Front. Microbiol..

[14] Krupovic M., Quemin E.R., Bamford D.H., Forterre P., Prangishvili D. (2013). Unification of the globally-distributed spindle-shaped viruses of archaea. J. Virol..

[15] Pietila M.K., Atanasova N.S., Oksanen H.M., Bamford D.H. (2013). Modified coat protein forms the flexible spindle-shaped virion of haloarchaeal virus His1. Environ. Microbiol..

[16] Bath C., Dyall-Smith M.L. (1998). His1, an archaeal virus of the Fuselloviridae family that infects *Haloarcula hispanica*. J. Virol..

[17] Demina T.A., Oksanen H.M. (2020). Pleomorphic archaeal viruses: The family Pleolipoviridae is expanding by seven new species. Arch. Virol..

[18] Lee S.T.M., Ding J.Y., Chiang P.W., Dyall-Smith M., Tang S.L. (2020). Insights into gene regulation of the halovirus His2 infecting *Haloarcula hispanica*. Microbiologyopen.

[19] Porter K., Kukkaro P., Bamford J.K., Bath C., Kivela H.M., Dyall-Smith M.L., Bamford D.H. (2005). SH1: A novel, spherical halovirus isolated from an australian hypersaline lake. Virology.

[20] Porter K., Russ B.E., Yang J., Dyall-Smith M.L. (2008). The transcription programme of the protein-primed halovirus SH1. Microbiology.

[21] Nuttall S.D., Dyall-Smith M.L. (1995). Halophage HF2: Genome organization and replication strategy. J. Virol..

[22] Dyall-Smith M., Tang S.L., Russ B., Chiang P.W., Pfeiffer F. (2020). Comparative genomics of two new HF1-like haloviruses. Genes.

[23] Dyall-Smith M.L. The Halohandbook: Protocols for Halobacterial Genetics. http://www.haloarchaea.com/resources/halohandbook/.

[24] Geneious. https://www.geneious.com/geneious/.

[25] Kearse M., Moir R., Wilson A., Stones-Havas S., Cheung M., Sturrock S., Buxton S., Cooper A., Markowitz S., Duran C. (2012). Geneious basic: An integrated and extendable desktop software platform for the organization and analysis of sequence data. Bioinformatics.

[26] Lomsadze A., Gemayel K., Tang S., Borodovsky M. (2018). Modeling leaderless transcription and atypical genes results in more accurate gene prediction in prokaryotes. Genome Res..

[27] Delcher A.L., Bratke K.A., Powers E.C., Salzberg S.L. (2007). Identifying bacterial genes and endosymbiont DNA with Glimmer. Bioinformatics.

[28] Noe L., Kucherov G. (2005). Yass: Enhancing the sensitivity of DNA similarity search. Nucleic Acids Res..

[29] Yass Genomic Similarity Search Tool. http://bioinfo.lifl.fr/yass/index.php.

[30] Genewiz Browser. http://www.cbs.dtu.dk/services/gwBrowser/.

[31] Img/vr Spacer Blast Tool. https://img.jgi.doe.gov/cgi-bin/vr),.

[32] CRISPRs Web Server. http://crispr.i2bc.paris-saclay.fr/.

[33] VIRFAM, Remote Homology Detection of Viral Protein Families. http://biodev.cea.fr/virfam/.

[34] Lopes A., Tavares P., Petit M.A., Guerois R., Zinn-Justin S. (2014). Automated classification of tailed bacteriophages according to their neck organization. BMC Genomics.

[35] Phobius A Combined Transmembrane Topology and Signal Peptide Predictor. https://phobius.sbc.su.se/.

[36] Kall L., Krogh A., Sonnhammer E.L. (2007). Advantages of combined transmembrane topology and signal peptide prediction--the Phobius web server. Nucleic Acids Res..

[37] Becker E.A., Seitzer P.M., Tritt A., Larsen D., Krusor M., Yao A.I., Wu D., Madern D., Eisen J.A., Darling A.E. (2014). Phylogenetically driven sequencing of extremely halophilic archaea reveals strategies for static and dynamic osmo-response. PLoS Genet..

[38] Nuttall S.D., Dyall-Smith M.L. (1993). Ch2, a novel halophilic archaeon from an australian solar saltern. Int. J. Syst. Bacteriol..

[39] REBASE The Restriction Enzyme Database. http://rebase.neb.com/rebase/rebase.html.

[40] Moraru C., Varsani A., Kropinski A.M. (2020). VIRIDIC-a novel tool to calculate the intergenomic similarities of prokaryote-infecting viruses. Viruses.

[41] Barylski J., Enault F., Dutilh B.E., Schuller M.B., Edwards R.A., Gillis A., Klumpp J., Knezevic P., Krupovic M., Kuhn J.H. (2020). Analysis of spounaviruses as a case study for the overdue reclassification of tailed phages. Syst. Biol..

[42] Meier-Kolthoff J.P., Goker M. (2017). VICTOR: Genome-based phylogeny and classification of prokaryotic viruses. Bioinformatics.

[43] Jamet A., Touchon M., Ribeiro-Goncalves B., Carrico J.A., Charbit A., Nassif X., Ramirez M., Rocha E.P.C. (2017). A widespread family of polymorphic toxins encoded by temperate phages. BMC Biol..

[44] Xu J., Hendrix R.W., Duda R.L. (2004). Conserved translational frameshift in dsDNA bacteriophage tail assembly genes. Mol. Cell.

[45] Xu J., Hendrix R.W., Duda R.L. (2014). Chaperone-protein interactions that mediate assembly of the bacteriophage lambda tail to the correct length. J. Mol. Biol..

[46] Mahony J., Alqarni M., Stockdale S., Spinelli S., Feyereisen M., Cambillau C., Sinderen D.V. (2016). Functional and structural dissection of the tape measure protein of lactococcal phage TP901-1. Sci. Rep..

[47] Tebbe A., Klein C., Bisle B., Siedler F., Scheffer B., Garcia-Rizo C., Wolfertz J., Hickmann V., Pfeiffer F., Oesterhelt D. (2005). Analysis of the cytosolic proteome of *Halobacterium salinarum* and its implication for genome annotation. Proteomics.

[48] Murphy J., Bottacini F., Mahony J., Kelleher P., Neve H., Zomer A., Nauta A., van Sinderen D. (2016). Comparative genomics and functional analysis of the 936 group of lactococcal *Siphoviridae* phages. Sci. Rep..

[49] Wyatt H.D., West S.C. (2014). Holliday junction resolvases. Cold Spring Harb. Perspect. Biol..

[50] Ennifar E., Basquin J., Birkenbihl R., Suck D. (2005). Purification, crystallization and preliminary x-ray diffraction studies of the archaeal virus resolvase SIRV2. Acta Crystallogr. Sect. F Struct. Biol. Cryst. Commun..

[51] Whittaker C.A., Hynes R.O. (2002). Distribution and evolution of von Willebrand/integrin a domains: Widely dispersed domains with roles in cell adhesion and elsewhere. Mol. Biol. Cell.

[52] Wong K.S., Houry W.A. (2012). Novel structural and functional insights into the MoxR family of AAA+ ATPases. J. Struct. Biol..

[53] Snider J., Houry W.A. (2006). MoxR AAA+ ATPases: A novel family of molecular chaperones?. J. Struct. Biol..

[54] Scheele U., Erdmann S., Ungewickell E.J., Felisberto-Rodrigues C., Ortiz-Lombardia M., Garrett R.A. (2011). Chaperone role for proteins p618 and p892 in the extracellular tail development of Acidianus two-tailed virus. J. Virol..

[55] Tsai Y.C., Ye F., Liew L., Liu D., Bhushan S., Gao Y.G., Mueller-Cajar O. (2020). Insights into the mechanism and regulation of the CbbQO-type rubisco activase, a MoxR AAA+ ATPase. Proc. Natl. Acad. Sci. USA.

[56] Wong K.S., Bhandari V., Janga S.C., Houry W.A. (2017). The RavA-ViaA chaperone-like system interacts with and modulates the activity of the fumarate reductase respiratory complex. J. Mol. Biol..

[57] Krishna S.S., Majumdar I., Grishin N.V. (2003). Structural classification of zinc fingers: Survey and summary. Nucleic Acids Res..

[58] To K.H., Young R. (2014). Probing the structure of the S105 hole. J. Bacteriol..

[59] Cahill J., Young R. (2019). Phage lysis: Multiple genes for multiple barriers. Adv. Virus Res..

[60] Casjens S.R., Gilcrease E.B., Winn-Stapley D.A., Schicklmaier P., Schmieger H., Pedulla M.L., Ford M.E., Houtz J.M., Hatfull G.F., Hendrix R.W. (2005). The generalized transducing *Salmonella* bacteriophage ES18: Complete genome sequence and DNA packaging strategy. J. Bacteriol..

[61] Desiere F., Mahanivong C., Hillier A.J., Chandry P.S., Davidson B.E., Brussow H. (2001). Comparative genomics of lactococcal phages: Insight from the complete genome sequence of *Lactococcus lactis* phage BK5-T. Virology.

[62] Mizuno C.M., Rodriguez-Valera F., Garcia-Heredia I., Martin-Cuadrado A.B., Ghai R. (2013). Reconstruction of novel cyanobacterial siphovirus genomes from mediterranean metagenomic fosmids. Appl. Environ. Microbiol..

[63] Millard A.D., Pearce D., Zwirglmaier K. (2016). Biogeography of bacteriophages at four hydrothermal vent sites in the Antarctic based on g23 sequence diversity. FEMS Microbiol. Lett..

[64] DSMZ Webserver (VICTOR). https://victor.dsmz.de.

[65] Robinson C.K., Wierzchos J., Black C., Crits-Christoph A., Ma B., Ravel J., Ascaso C., Artieda O., Valea S., Roldan M. (2015). Microbial diversity and the presence of algae in halite endolithic communities are correlated to atmospheric moisture in the hyper-arid zone of the Atacama desert. Environ. Microbiol..

[66] Wang J., Liu Y., Liu Y., Du K., Xu S., Wang Y., Krupovic M., Chen X. (2018). A novel family of tyrosine integrases encoded by the temperate pleolipovirus SNJ2. Nucleic Acids Res..

[67] Gcf_000337035.1 (*Hrr. coriense* Genome Assembly). https://www.ncbi.nlm.nih.gov/assembly/GCF_000337035.1/.

[68] Pagaling E., Haigh R.D., Grant W.D., Cowan D.A., Jones B.E., Ma Y., Ventosa A., Heaphy S. (2007). Sequence analysis of an archaeal virus isolated from a hypersaline lake in Inner Mongolia, China. BMC Genomics.

[69] Podell S., Ugalde J.A., Narasingarao P., Banfield J.F., Heidelberg K.B., Allen E.E. (2013). Assembly-driven community genomics of a hypersaline microbial ecosystem. PLoS ONE.

[70] Bath C., Cukalac T., Porter K., Dyall-Smith M.L. (2006). His1 and His2 are distantly related, spindle-shaped haloviruses belonging to the novel virus group, Salterprovirus. Virology.

[71] Liu Y., Wang J., Liu Y., Wang Y., Zhang Z., Oksanen H.M., Bamford D.H., Chen X. (2015). Identification and characterization of SNJ2, the first temperate pleolipovirus integrating into the genome of the SNJ1-lysogenic archaeal strain. Mol. Microbiol..

[72] Demina T.A., Atanasova N.S., Pietila M.K., Oksanen H.M., Bamford D.H. (2016). Vesicle-like virion of Haloarcula hispanica pleomorphic virus 3 preserves high infectivity in saturated salt. Virology.

[73] Dyall-Smith M., Tang S.-L., Pfeiffer F. (2019). Haloviruses: The bad, the worse and the surprising. Studia Universitatis Babes-Bolyai, Biologia.

[74] Schulze S., Adams Z., Cerletti M., De Castro R., Ferreira-Cerca S., Fufezan C., Gimenez M.I., Hippler M., Jevtic Z., Knuppel R. (2020). The archaeal proteome project advances knowledge about archaeal cell biology through comprehensive proteomics. Nat. Commun..

[75] Esquivel R.N., Schulze S., Xu R., Hippler M., Pohlschroder M. (2016). Identification of *Haloferax volcanii* pilin N-glycans with diverse roles in pilus biosynthesis, adhesion, and microcolony formation. J. Biol. Chem..

[76] Laass S., Monzon V.A., Kliemt J., Hammelmann M., Pfeiffer F., Forstner K.U., Soppa J. (2019). Characterization of the transcriptome of *Haloferax volcanii*, grown under four different conditions, with mixed RNA-seq. PLoS ONE.

[77] Kucukyildirim S., Behringer M., Williams E.M., Doak T.G., Lynch M. (2020). Estimation of the genome-wide mutation rate and spectrum in the archaeal species *Haloferax volcanii*. Genetics.

[78] Collins M., Afolayan S., Igiraneza A.B., Schiller H., Krespan E., Beiting D.P., Dyall-Smith M., Pfeiffer F., Pohlschroder M. (2020). Mutations affecting HVO_1357 or HVO_2248 cause hypermotility in *Haloferax volcanii*, suggesting roles in motility regulation. Genes.

[79] Dyall-Smith M., Palm P., Wanner G., Witte A., Oesterhelt D., Pfeiffer F. (2019). *Halobacterium salinarum* virus ChaoS9, a novel halovirus related to phih1 and phich1. Genes.

[80] Fullmer M.S., Ouellette M., Louyakis A.S., Papke R.T., Gogarten J.P. (2019). The patchy distribution of restriction-modification system genes and the conservation of orphan methyltransferases in halobacteria. Genes.

[81] Tang S.L., Nuttall S., Ngui K., Fisher C., Lopez P., Dyall-Smith M. (2002). HF2: A double-stranded DNA tailed haloarchaeal virus with a mosaic genome. Mol. Microbiol..

[82] Russ B. (2009). Unravelling the transcriptional programme of the haloarchaeal virus HF2.

[83] Zecchi L., Lo Piano A., Suzuki Y., Canas C., Takeyasu K., Ayora S. (2012). Characterization of the Holliday junction resolving enzyme encoded by the *Bacillus subtilis* bacteriophage spp1. PLoS ONE.

[84] Birkenbihl R.P., Neef K., Prangishvili D., Kemper B. (2001). Holliday junction resolving enzymes of archaeal viruses SIRV1 and SIRV2. J. Mol. Biol..

[85] Kaur G., Subramanian S. (2016). Classification of the treble clef zinc finger: Noteworthy lessons for structure and function evolution. Sci. Rep..

[86] Nagel C., Machulla A., Zahn S., Soppa J. (2019). Several one-domain zinc finger micro-proteins of *Haloferax volcanii* are important for stress adaptation, biofilm formation, and swarming. Genes.

