# Supplementary material for: The Novel Halovirus Hardycor1, and the Presence of Active (Induced) Proviruses in Four Haloarchaea
Source: Genes (Basel). 2021 Jan 23;12(2):149. doi: 10.3390/genes12020149 (PMC7911831; doi:10.3390/genes12020149)
Supplement: Supplementary file 1 [file genes-12-00149-s001.pdf]

Supplementary Materials.

Table S1. DNA methyltransferases and restriction endonucleases of *Hrr. coriense* Ch2^{T} (DSM 10284).

**Locus_tag**[a]	**Annotated product**[b]
C464_00174	Site-specific DNA-cytosine methylase (COG0270)
C464_04156	Site-specific DNA adenine methylase (COG0338)
C464_05570	Mrr restriction endonuclease (COG1715)
C464_16257	Mrr restriction endonuclease (COG1715)
C464_16637	Site-specific DNA-cytosine methyltransferase (COG0270)

[a]Genbank accession AOJL00000000.1

[b]Mrr, Methylated adenine Recognition and Restriction system.

Table S2. VICTOR[a] prediction of taxonomic classification of tailed haloviruses

	DNA (D_0)			Protein (D_6)		
**TAILED HALOVIRUS**	**Species**	**Genus**	**Family**	**Species**	**Genus**	**Family**
BJ1 virus (AM419438)	1	1	1	1	1	1
CGphi46 (HQ332141)	2	1	1	2	1	1
HSTV-1 (KC117378)	3	2	1	3	2	1
HSTV-2 (KC117376)	4	3	1	4	3	2
HRTV-7 (KC292021)	5	3	1	4	3	2
HHTV-1 (KC292025)	6	4	2	5	4	1
HGTV-1 (KC292026)	7	5	1	6	5	2
HHTV-2 (KC292024)	8	6	1	7	6	2
HCTV-2 (KC292028)	9	6	1	8	6	2
HVTV-1 (KC117377)	10	7	1	9	7	2
HCTV-5 (KC292027)	11	7	1	10	7	2
HCTV-1 (KC292029)	12	7	1	11	7	2
HRTV-4 (KC292023)	13	8	1	12	8	1
HFTV-1 (MG550112)	14	8	1	13	8	1
phiH1 (MK002701)	15	9	1	14	9	1
ChaoS9 (MK310226)	16	9	1	15	9	1
phiCh1 (MK450543)	17	9	1	16	9	1
HF2 (AF222060)	18	10	1	17	3	2
HF1 (AY190604)	19	10	1	17	3	2
HRTV-8 (KC292020)	20	10	1	18	3	2
HRTV-5 (KC292022)	21	10	1	17	3	2
Hardycor2 (MN901520)	22	10	1	17	3	2
Serpecor1 (MN901521)	23	10	1	19	3	2
**Hardycor1** (MT152698)	24	11	1	20	10	1

[a] as implemented at the DSMZ webserver https://victor.dsmz.de [1].

Table S3: CRISPR spacer match to Hardycor1

Start (nt)	Alignment[a]	End (nt)	Identical nt (E-value)
Hardycor1 17635<br>Ga0268433 40	TACACGAGGCCGGCTACGACTACGAGACGATCCCCAAACT<br>..................................AC....	17674<br>1	38/40nt<br>(E= 2 x 10^{-7})

Details of the Ga0268433 CRISPR spacer

ID	Scaffold:Ga0268433_1033988<br>Contig: 3300027982:Ga0268433_1033988:1:1206
Sample source	Halite endolithic microbial communities from Salar Grande, Atacama Desert, Chile.
DR of CRISPR	GTTTCAATCCCGTCCTGGGTTTTCTCCCT
Most similar DR	*Halostagnicola larsenii* XH-48(NZ_CP007056) gtttcaatcccgttctgggttttctc

[a] Dots indicate identical bases to the Hardycor1 sequence.

[b]The IMG/VR website (https://img.jgi.doe.gov/index.html) was accessed December 5, 2020

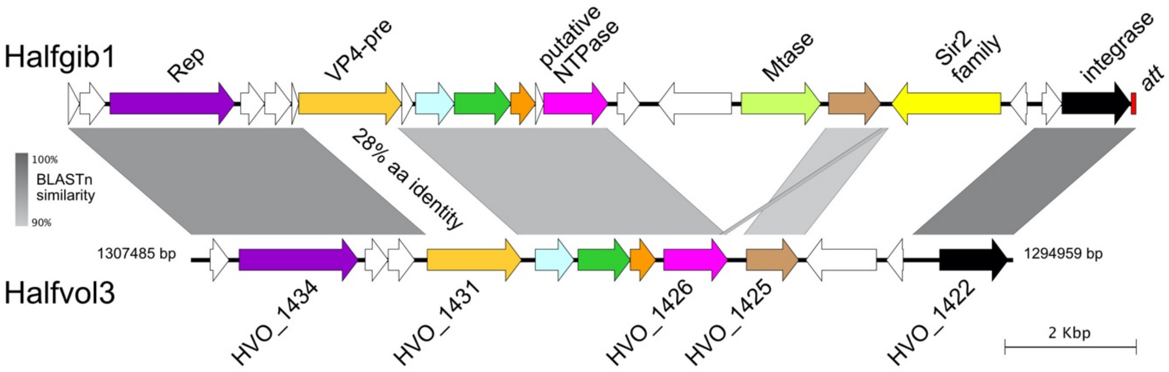

Figure S1. Comparison of the genomes of Halfgib1 and Halfvol3 proviruses. Halfgib1 is located between nt 269983-286444 on contig AOLJ01000022 of the *Hfx. gibbonsii* Ma2.38^{T} genome. It is found integrated at tRNA-Arg (C454_t15621). Several genes have been labeled according to their similarity to annotated proteins of pleolipoviruses, such as HRPV1. Rep, replication protein; VP4-pre, virus protein VP4 precursor; Mtase, methyltransferase; Sir2 family, sirtuin family protein; *att*, attachment sequence for integration. BLASTn similarity between the two proviruses is indicated by shading (key given at left edge). Halfvol3 is located on the chromosome of *Hfx. volcanii* DS2 (nt 1294959-1307485). Size scale shown at lower right.

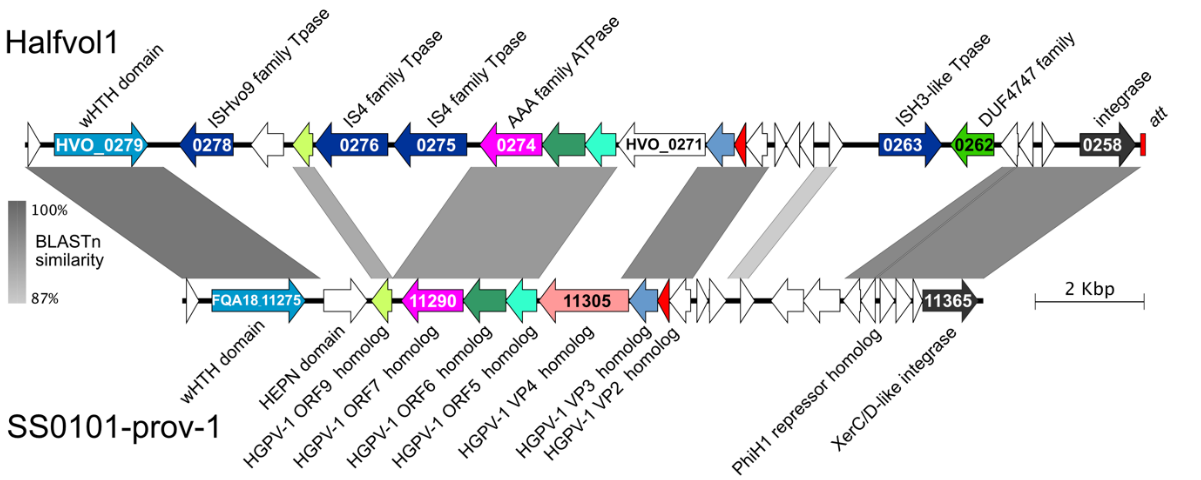

Figure S2. Comparison of the genomes of proviruses Halfvol1 and SS0101-prov1. Halfvol1 is 20,715 bp (*Hfx. volcanii* DS2^{T}, nt 231452–252038; CP001956), and is shown from HVO_0280A (left end) to HVO_0258 (integrase, right end). In the provirus state it is integrated at tRNA-Pro (HVO_3017). The provirus of *Hfx. volcanii* strain SS0101 is 14,675 bp is found integrated in the corresponding tRNA-Pro (FQA18_19160) of that genome (accession VMTR00000000.1). SS0101-prov1 is found on contig VMTR01000075 and spans from FQA18_11270 (left end) to the integrase (FQA18_11365, right end). Nucleotide similarity (BLASTn, E-value ≤ 10^{-20}) between the two proviruses is indicated by grey shading (key at left edge). Both proviruses are related to betapleolipoviruses, as is indicated by the labeling of many SS0101-prov-1 genes as encoding proteins that show strong protein similarity to corresponding proteins encoded by the betapleolipovirus HGPV-1 [2]. A size scale is shown at the right. Several key genes and locus_tags are indicated within gene arrows.

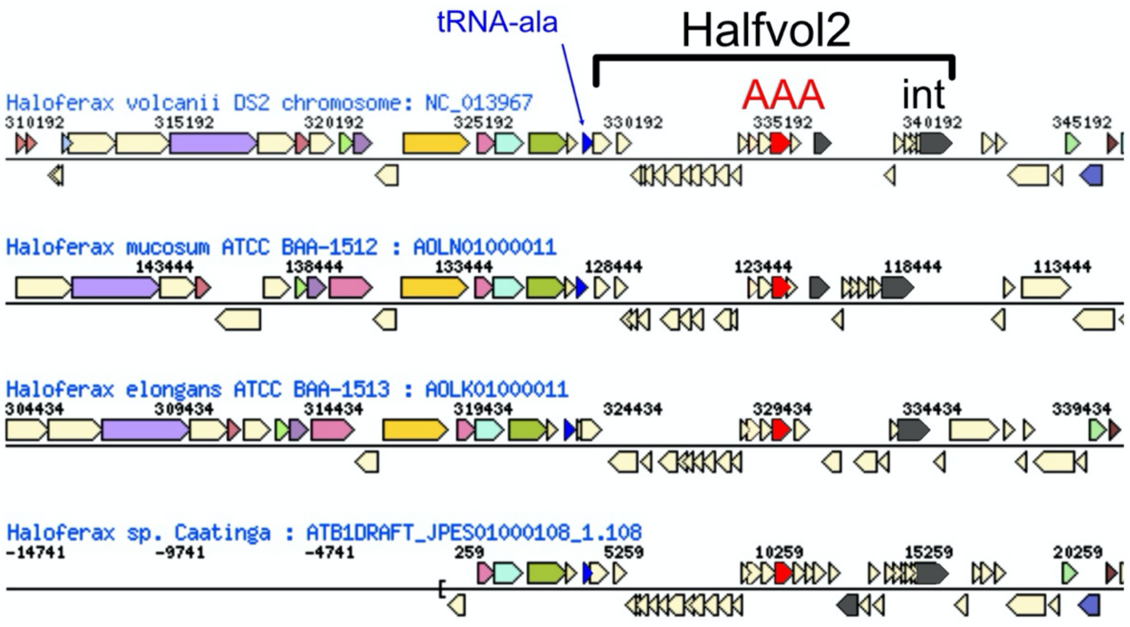

Figure S3. Gene Ortholog Neighbourhoods of Halfvol2 aligned using HVO_2248 of *Hfx. volcanii* DS2 as the reference. Halfvol2-related provirus elements in the genomes of other *Haloferax* spp. were aligned using the "Show neighborhood regions with this gene's bidirectional best hits" option available at the DOE JGI IMG/MER website [3-5]. The tRNA-ala gene (HVO_3020, blue) is indicated by an arrow at the left of Halfvol2. The AAA-ATPase gene (HVO_0377, red) is labeled AAA, and the integrase gene at the other end of the element (HVO_0385, black) is labeled (int). In the other genomes below, the labeled genes of Halfvol2 have been coloured the same as in strain DS2.

Table S4. Sequence reads spanning the circularized termini of the three chromosomal proviruses (Halfvol1, Halfvol2 and Halfvol3) of *Hfx. volcanii* DS2.

A. SRX8436478 reads mapped to *Hfx. volcanii* DS2 or circularised proviruses using the Geneious mapping tool[a].

**Reads Spanning**	**Halfvol1**	**Halfvol2**	**Halfvol3**
Circularised provirus ends	2	0	12
Integrated provirus ends	158	160	148

[a]Sequence Read Archive (SRA), run SRR11888928, paired Illumina HiSeq 2500 (https://www.ncbi.nlm.nih.gov/sra/SRX8436478). Average read coverage = 160x.

B. SRX8436462 reads mapped to *Hfx. volcanii* DS2 or circularised proviruses using the Geneious mapping tool[a].

**Reads Spanning**	**Halfvol1**	**Halfvol2**	**Halfvol3**
Circularised provirus	170	0	3
Integrated provirus	40	56	32

[a]Sequence Read Archive (SRA), https://www.ncbi.nlm.nih.gov/sra/SRX8436462. Paired Illumina HiSeq 2500. Average read coverage = 102x, except across Halfvol1, where it is much higher, between 300-400x.

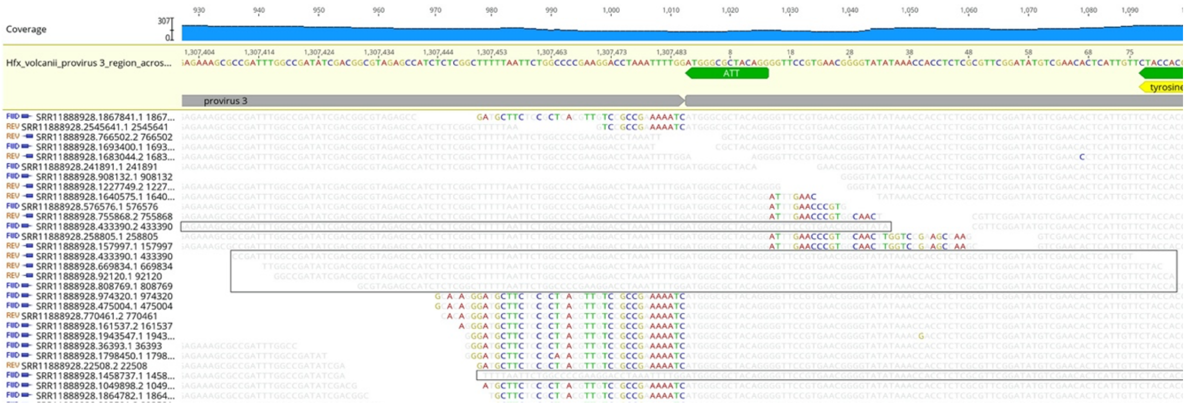

Figure S4. Screenshot of Geneious window showing *Hfx. volcanii* Hv90 illumina reads (SRA accession SRX8436478) mapped to the termini of circularised Halfvol3 (top sequence, with coloured bases). The position of the *att* sequence is shown by the green arrow. Reads matching the circularised provirus are boxed and have no coloured bases (no disagreements to the reference sequence). Reads matching the integrated provirus show base disagreements to Halfvol3 (coloured bases) that occur after the *att* region (green arrow at top).

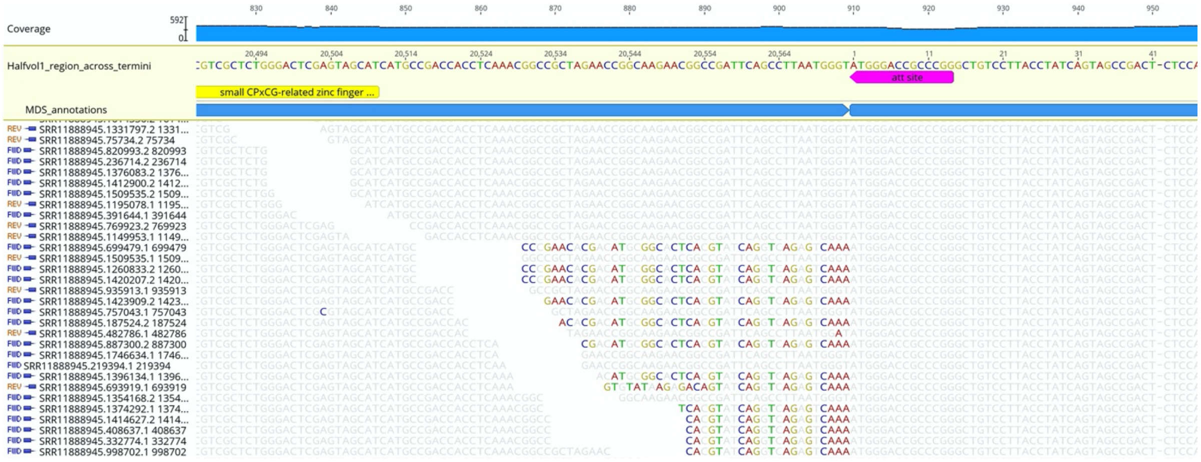

Figure S5. Screenshot of Geneious window showing *Hfx. volcanii* Hv1 reads (SRX8436462) mapped to the termini of circularised Halfvol1. Reads matching the circularised provirus have no coloured bases (disagreements to the reference sequence). Reads matching the integrated provirus show base disagreements to Halfvol1 (coloured bases) that occur after the *att* region (green arrow at top).

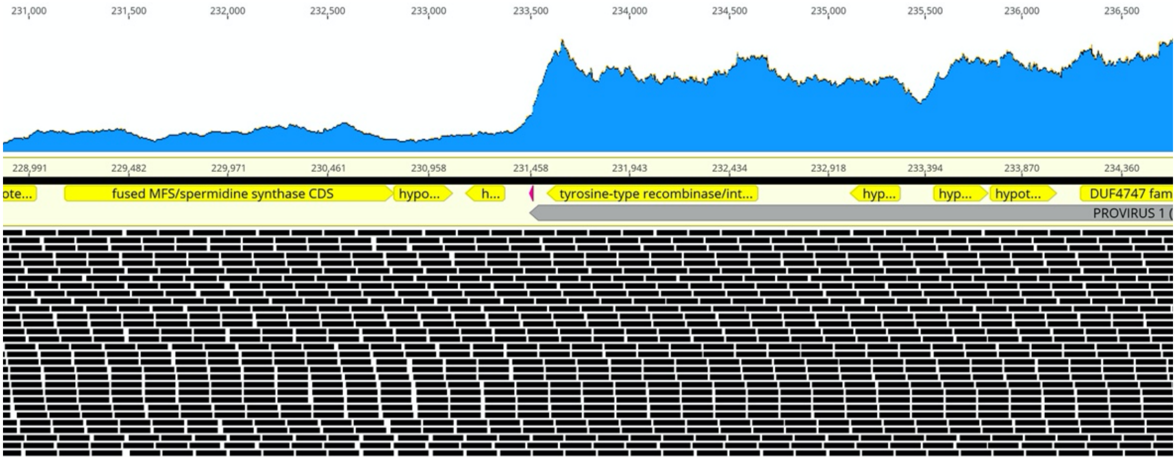

Figure S6. Screenshot of Geneious window showing *Hfx. volcanii* Hv1 reads (SRX8436462) mapped to *Hfx. volcanii* DS2, showing the region around the left end of provirus Halfvol1 on the main chromosome (CP001956). The read coverage is plotted in blue at the top, and rises dramatically at the provirus border from around 102 x outside of Halfvol1 to 300-400x within Halfvol1.

References

1. Meier-Kolthoff, J.P.; Goker, M. VICTOR: genome-based phylogeny and classification of prokaryotic viruses. *Bioinformatics* **2017**, *33*, 3396-3404.
2. Atanasova, N.S.; Demina, T.A.; Krishnam Rajan Shanthi, S.N.V.; Oksanen, H.M.; Bamford, D.H. Extremely halophilic pleomorphic archaeal virus HRPV9 extends the diversity of pleolipoviruses with integrases. *Res. Microbiol.* **2018**, *169*, 500-504.
3. Chen, I.A.; Chu, K.; Palaniappan, K.; Pillay, M.; Ratner, A.; Huang, J.; Huntemann, M.; Varghese, N.; White, J.R.; Seshadri, R., *et al.* IMG/M v.5.0: an integrated data management and comparative analysis system for microbial genomes and microbiomes. *Nucleic Acids Res.* **2019**, *47*, D666-D677.
4. Mukherjee, S.; Stamatis, D.; Bertsch, J.; Ovchinnikova, G.; Katta, H.Y.; Mojica, A.; Chen, I.A.; Kyrpides, N.C.; Reddy, T. Genomes OnLine database (GOLD) v.7: updates and new features. *Nucleic Acids Res.* **2019**, *47*, D649-D659.
5. JGI IMG/MER integrated microbial genomes and microbiomes. Available online: https://img.jgi.doe.gov/ (Accessed 1/6/2020).
